# Comparative genomic and transcriptomic analyses of African swine fever virus strains

**DOI:** 10.1016/j.csbj.2023.08.028

**Published:** 2023-08-29

**Authors:** Peng Lu, Jiaqiao Zhou, Sibo Wei, Konosuke Takada, Hayato Masutani, Suguru Okuda, Ken Okamoto, Michio Suzuki, Tomoya Kitamura, Kentaro Masujin, Takehiro Kokuho, Hideaki Itoh, Koji Nagata

**Affiliations:** a Department of Applied Biological Chemistry, Graduate School of Agricultural and Life Science, The University of Tokyo, 1-1-1 Yayoi, Bunkyo-ku, Tokyo 113-8657, Japan; b African Swine Fever Unit, National Institute of Animal Health, National A griculture and Food Research Organization (NARO), 6-20-1 Josuihoncho, Kodaira, Tokyo, Japan; c Agricultural Bioinformatics Research Unit, Graduate School of Agricultural and Life Science, The University of Tokyo, 1-1-1 Yayoi, Bunkyo-ku, Tokyo 113-8657, Japan

**Keywords:** African swine fever, Open reading frames, Broad-spectrum defense

## Abstract

African swine fever (ASF) is the most devastating disease caused by the African swine fever virus (ASFV), impacting the pig industry worldwide and threatening food security and biodiversity. Although two vaccines have been approved in Vietnam to combat ASFV, the complexity of the virus, with its numerous open reading frames (ORFs), necessitates a more diverse vaccine strategy. Therefore, we focused on identifying and investigating the potential vaccine targets for developing a broad-spectrum defense against the virus. This study collected the genomic and/or transcriptomic data of different ASFV strains, specifically from *in vitro* studies, focusing on comparisons between genotypes I, II, and X, from the National Center for Biotechnology Information (NCBI) database. The comprehensive analysis of the genomic and transcriptomic differences between high- and low-virulence strains revealed six early genes, 13 late genes, and six short genes as potentially essential ORFs associated with high-virulence. In addition, many other ORFs (*e.g.,* 14 multigene family members) are worth investigating. The results of this study provided candidate ORFs for developing ASF vaccines and therapies.

## Graphical Abstract

**Figure:**
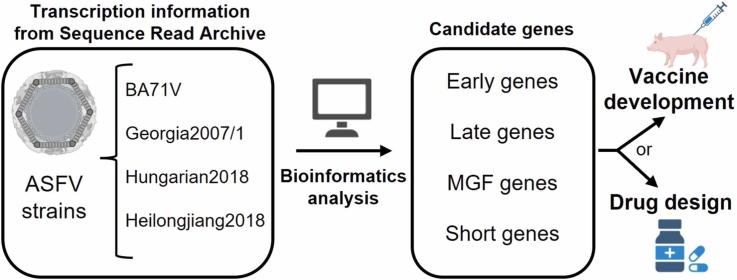

## 1 Introduction

African swine fever (ASF) is a devastating hemorrhagic viral disease affecting domestic and wild pigs of all age groups. According to retrospective data, Montgomery first documented ASF in 1921 in Kenya [1]. ASF was first introduced in Georgia in 2007 and has spread to European, Asian, Pacific, and Caribbean countries. As of July 2023, ASF has affected 18 countries in the Asian and Pacific region, with specific outbreaks, such as 36 domestic pig farms in Korea, 67 provinces in the Philippines, and 114 outbreaks in Thailand, thereby significantly impacting the pig industry [2].

ASF is caused by the African swine fever virus (ASFV) that is an icosahedral DNA virus with a diameter of 200 nm, composed of an envelope, capsid, inner capsule membrane, core shell, and inner core. A linear double-stranded DNA, ranging between 170 and 190 kbp depending on the isolate, was encapsulated in its inner core, encoding 150–200 viral proteins. These proteins consist of 68 structural and more than 100 non-structural proteins [3].

Furthermore, no effective antiviral drugs were available to prevent or treat ASF. However, in August 2022, the US Department of Agriculture released one vaccine (NAVET–ASFVAC) by the National Veterinary Medicine Joint Stock Company [4]. This vaccine is based on an attenuated virus strain ASFV-G-ΔI177L, which targets the open reading frame (ORF), I177L. It is transcribed as a late gene during the virus replication cycle and encodes a 177-amino-acid protein containing a possible N-terminal transmembrane helix. The specific function of this protein is not well-defined in the literature, but it is recognized as a viral genetic determinant of virulence [5].

Another vaccine, “AVAC,” developed and produced by AVAC Vietnam Co. Ltd., has been approved for use under supervision in Vietnam. This vaccine is based on the ASFV-G-ΔMGF strain, where specific MGF genes have been deleted, but the exact details have not been disclosed [6].

Despite these significant advances, relying on one or two vaccines is insufficient to completely eradicate the virus, especially for viruses, like ASFV with many ORFs. It is well-understood that viruses with high mutation rates, such as the estimated substitution rate of approximately 6.7 × 10^−4^ substitutions per site per year for ASFV, and complex interactions with host immune systems, can escape or become resistant to the effects of a single vaccine [7]. Therefore, our efforts for eradicating ASFV should not halt the development of two vaccines but explore other potential targets for vaccine and drug development. Given the abundant ORFs present in ASFV, other ORFs could likely serve as potential targets. Hence, to enhance our defenses against ASFV, it is crucial to develop broader therapies targeting multiple ORFs, which could offer more comprehensive protection against this complex and adaptable virus.

Vaccines and antivirals that can work on multiple targets will have higher chances of success. Hence, it is essential to screen candidate ORFs other than I177L. More than 170 genomes of ASFV strains have been sequenced. The virulence of ASFV strains is highly dependent on their ORFs. Pig/HLJ/2018 (HLJ2018) from China, ASFV_HU_2018 (HG2018) from Hungary, and Georgia 2007/1 (GRG2007) from Georgia are highly virulent genotype II strains, whereas OURT 88/3 and BA71V are avirulent genotype I strains. Genome alignments and strain comparisons have been well documented [8], and several studies have focused on the transcriptional level of one or two ASFV strains [9], [10], [11]. However, comparative analyses of multiple ASFV strains at the transcriptional level are still scarce.

Our primary hypothesis is that distinct patterns of ORF expression can be associated with the virulence of ASFV strains. Therefore, this study identified genomic and transcriptomic differences between high- and low-virulence strains. The results obtained in this analysis provided novel concepts and a theoretical basis for selecting ORF candidates for vaccine/drug development.

## 2 Methods

### 2.1 Genomic comparison

The genomic sequences of BA71V (NC_001659.2), OURT88/3 (NC_044957.1), HLJ2018 (MK333180.1), Hungarian2018 (MN715134.1), and Georgia2007/1 (NC_044959.2) were obtained from the National Center for Biotechnology Information (NCBI). The genomic sequence alignment of each strain was performed using Proksee (proksee.*ca*) [12].

### 2.2 RNA-seq data collection

The data objects were obtained from the Sequence Read Archive (SRA) database, NCBI by sra-tools. The sra files, returned by the ‘prefetch’ function, were converted to a fastq format using the ‘fasterq-dump’ function. The details of the selected high-throughput sequencing data of mRNA are listed in Table 1 [9], [13], [14], [15]. A post-infection time at or shorter than 6 h was defined as the early stage, and a post-infection time at or longer than 12 h was defined as the late stage.

**Table 1:** Information of the collected data.

Strain	Infected target/sample	Multiplicity of infection (MOI)	Early stage (< 6 h)	Late stage (> 12 h)	Reference
BA71V	Vero cells	5	SRR10504831 (5 h) SRR10504832 (5 h)	SRR10504829 (16 h) SRR10504830 (16 h)	Cackett et al., 2020
Heilongjiang2018	Porcine alveolar macrophages	1	SRR11185050 (6 h) SRR11185049 (6 h) SRR11185048 (6 h)	SRR11185053 (12 h) SRR11185052 (12 h) SRR11185051 (12 h)	Ju et al., 2021
Hungarian2018	Porcine alveolar macrophages	10	ERR3895479 (4 h) ERR3895483 (4 h)	ERR3895481 (12 h) ERR3895485 (12 h)	Olasz et al., 2020
Georgia2007/1	Porcine alveolar macrophages	5	SRR14862468 (5 h) SRR14862467 (5 h)	SRR14862466 (16 h) SRR14862465 (16 h)	Cackett et al., 2022

### 2.3 RNA-seq data processing and mapping

FastQC performed quality control checks on the raw sequence data. The mRNA reads were mapped to BA71V (NC_001659.2), HLJ2018 (MK333180.1), Hungarian2018 (MN715134.1), and Georgia2007/1 (NC_044959.2), respectively, by HISAT2 [16]. All annotation files obtained from the NCBI database were confirmed, and errors were fixed. The expression levels of mRNA were counted by featureCounts [17] using relative genome indices generated by HISAT2 [18].

### 2.4 Identifying differentially expressed genes (DEGs)

The expression matrices created by featureCounts were estimated by MultiQC [19] and analyzed using R software (version R 4.0.0) using the DESeq2 R package [20]. The fold change (FC) in the original data was log2 transformed, and log2FC shrinkage was performed by the apeglm [21] R package.

Significant DEGs between the early and late stages were determined using the threshold of Benjamini—Hochberg [22] adjusted *p-*value < 0.05 and log2FC shrinkage values > 1.0 or < –1.0. Log2FC shrinkage values > 1.0 were considered early genes, while log2FC shrinkage values < –1.0 were considered late genes.

The Pearson correlation coefficient (PCC) between each pair of DEGs was calculated. PCC > 0.7 and *P* < 0.001 were considered strong correlations.

The total number of mapped ASFV reads was summarized in Supplementary File 1. Despite the differences in the reads number, the heatmap of the expression of each ORF in different strains was created by the heatmap R package based on the normalized expression values in transcripts per million (TPM) of DEGs. The heatmap was generated based on the Z-score calculated from each ORF’s original TPM values in different ASFV strains. The Z-score calculation is performed column-wise (vertically) rather than row-wise (horizontally).

DEGs were visualized in the volcano plots using limma [23] and the ggplot2 [24] R package. Log10(TPM) ≤ 1 was defined as non-expressed ORFs.

### 2.5 Structure and interaction prediction of essential genes

AlphaFold ver2.2.0 predicted the complex structures of significant genes [25], [26]. The program was executed with the latest databases and the following parameters: --model_preset = multimer, db_preset = full_dbs, --use_gpu_relax = true, --num_multimer_predictions_per_model = 1, max_template_date = 2099–07–14.

Five models were generated for each complex, and their ranking was determined by the ranking_confidence score, a linear combination that amalgamates the interface score (ipTM) × 0.8 and the overall structural score (pTM) × 0.2. Subsequently, the model with the highest ranking_confidence score was selected for further analysis. A ranking confidence score of ≥ 0.7 was set as the threshold for a possible model confidence cut-off [27].

## 3 Results

### 3.1 Genome comparison

**Fig. 1:**
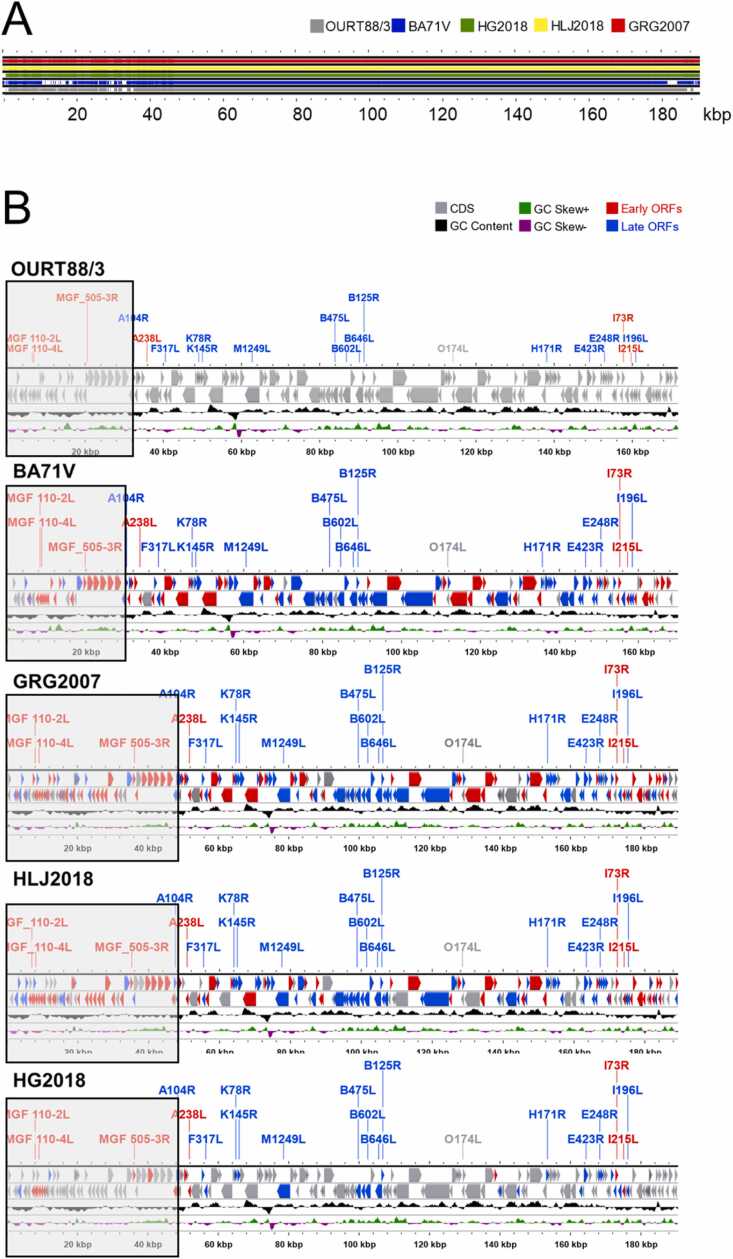
Genome sequence alignment and comparison of two low-virulence African swine fever virus (ASFV) strains (BA71V and OURT88/3) and three high-virulence ASFV strains (GRG2007, HG2018, and HLJ2018). A: Genome alignment. B: Comparison of open reading frames in different strains. Early, late, and constant genes are colored red, blue, and gray, respectively. Twenty common genes in each strain are labeled and colored.

Genome sequence alignments of OURT88/3, BA71V, GRG2007, HG2018, and HLJ2018 showed that highly virulent strains are similar in length (Fig. 1). The genomes of the avirulent strains (BA71V and OURT88/3) are ∼30 kbp shorter than those of the virulent strains because of three genetic deletions observed at approximately 15–20 kbp, ∼30 kbp, and ∼180 kbp regions (Fig. 1A). Due to the deletions, the number of multigene family (MGF) members in BA71V and OURT88/3 are less than in GRG2007, HG2018, and HLJ2018 (Fig. 1B).

**Fig. 2:**
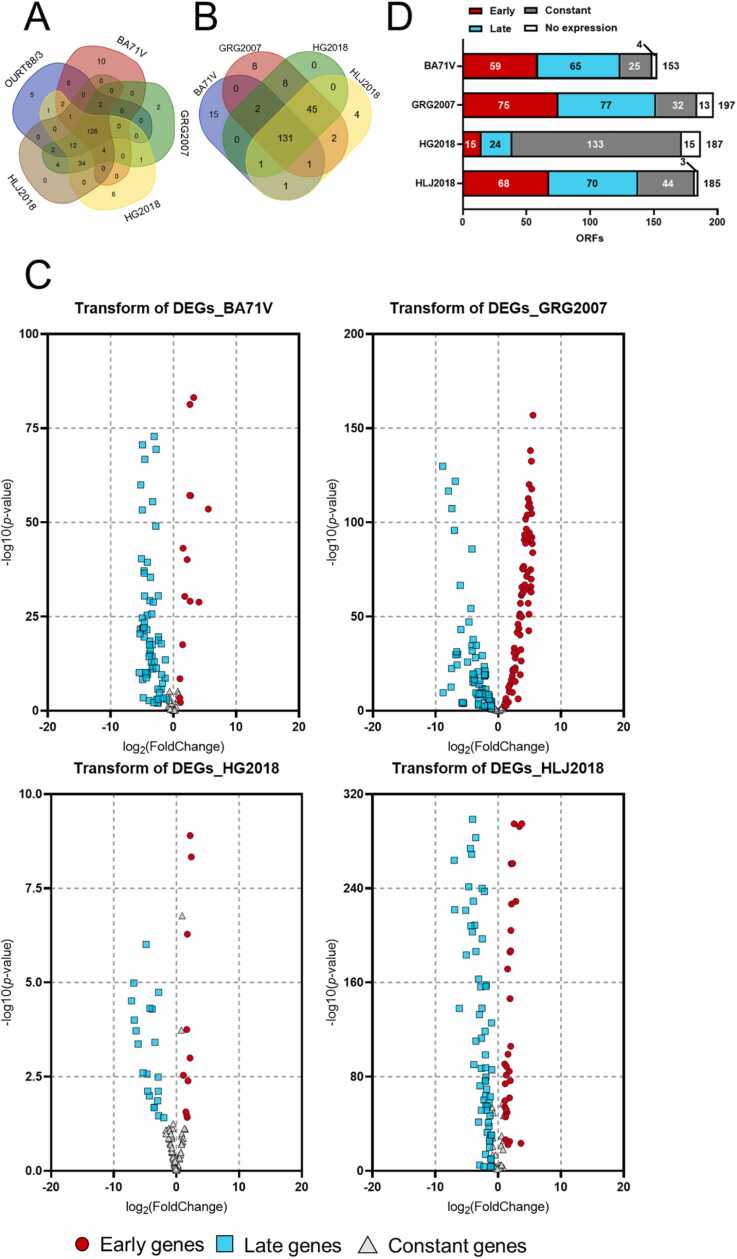
Venn diagrams and summary of open reading frames (ORFs). A: Venn diagram of ORFs from BA71V, OURT88/3, GRG2007, HG2018, and HLJ2018. B: Venn diagram of ORFs from BA71V, GRG2007, HG2018, and HLJ2018. C: Volcano plots of the ORFs from BA71V, GRG2007, HG2018, and HLJ2018. The vertical axis represented the significant difference between the early and late stages of African swine fever virus (ASFV) infection. The horizontal axis represented the fold change between the early and late stages of ASFV infection. Early, late, and constant genes are colored red, blue, and gray, respectively. D: Summary of early, late, constant, and non-expressed genes.

The comparisons of ORFs between the virulent (GRG2007, HG2018, and HLJ2018) and avirulent strains (BA71V and OURT88/3) showed that 126 ORFs are present in all five strains (BA71V, OURT88/3, GRG2007, HG2018, and HLJ2018) (Fig. 2A). When a comparison was performed without OURT88/3, 131 ORFs were present in four strains (Fig. 2B). Table 2 summarizes that 14 ORFs exist in all the virulent strains, which are MGF members. On the other hand, 19 ORFs are not present in any virulent strains but are present in avirulent strains (BA71V and OURT88/3), nine of which are MGF members. Interestingly, with the inclusion of an intermediate virulent strain (genotype X), Kenya05 (KM111294.1), similar results were obtained (Supplementary Table 1). Three ORFs are commonly present only in GRG2007, HG2018, and HLJ2018. Eleven ORFs are commonly present in GRG2007, HG2018, HLJ2018, and Kenya05. These ORFs are identical to the 14 ORFs in all virulent strains analyzed in Table 2. Among the 19 genes in the attenuated strain (Table 2), fifteen are identical in an intermediate virulent strain (Supplementary Table 1).

**Table 2:** Features of ORFs in virulent and avirulent strains.

ORFs only presented in all virulent strains (GRG2007, HG2018, HLJ2018)	ORFs only presented in either of the avirulent strains (BA71V, OURT88/3)
MGF_100-1L	86R
MGF_110-3L	EP36R
MGF_110-5L-6L	X64R
MGF_110-7L	KP86R
MGF_110-8L	C105R
MGF_110-12L	L57L
MGF_360-10L	DP86L
MGF_360-11L	DP93R
MGF_360-12L	J64R
MGF_360-13L	KP93L
MGF_360-14L	MGF_100-2L
MGF_360-21R	MGF_110-5L
MGF_505-1R	MGF_110-6L
MGF_505-6R	MGF_360-2LA
	MGF_360-16RA
	MGF_360-17R
	MGF_360-19R
	MGF_505-8R
	MGF_505-11R

The reduced ORFs, including MGF_100–2L and MGF_110–5L, are shared by BA71V, OURT88, and Kenya05; MGF_110–6L is shared by BA71V and Kenya05; OURT88 and Kenya05 share MGF_360–19R.

### 3.2 Expression comparison

As no RNA-seq data was available for OURT88/3 in the Sequence Read Archive (SRA) database, the following analysis was performed with the exclusion of OURT88/3 (Supplementary Table 1).

To evaluate the importance of each ORF in each strain, the expression levels at the early and late stages were compared in the same strain. The results were visualized as volcano plots (Fig. 2C). In BA71V, 153 ORFs are present in the genome. As a result, 59 and 65 ORFs were identified as early and late genes, respectively, whereas 25 ORFs were identified as constant genes. No expression was detected for four ORFs. In GRG2007, 197 ORFs are present in the genome. As a result, 75 and 77 ORFs were identified as early and late genes, respectively, whereas 32 genes were identified as constant. No expression was detected for 13 ORFs. In HG2018, 187 ORFs are present in the genome. As a result, 15 and 24 ORFs were identified as early and late genes, respectively, whereas 133 genes were identified as constant. No expression was detected for 16 ORFs. In HLJ2018, 185 ORFs are present in the genome. As a result, 68 and 70 ORFs were identified as early and late genes, respectively, whereas 44 genes were identified as constant. No expression was detected for three ORFs. These results are summarized in Fig. 2D**.** Interestingly, ORF I243L, an ORF that exhibited an 'early gene' in avirulent strains but was a 'late gene' in virulent ones. This distinct expression pattern may be critical for understanding the virulence of ASFV strains.

**Fig. 3:**
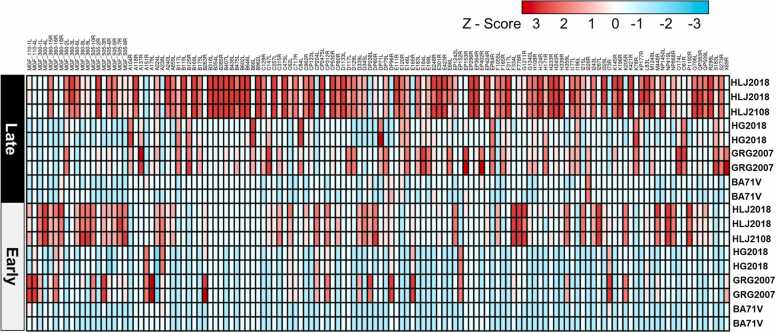
Heatmap representation of gene expression profiles across different African swine fever virus (ASFV) strains (BA71V, GRG2007, HG2018, and HLJ2018). The heatmap was generated using the Z-scores calculated from the transcript per million values of individual open reading frames (ORFs) across various ASFV strains. Each row represented a dataset of one ASFV strain, and each column represented a distinct ORF. The relative expression levels (Z-score) are calculated vertically (*i.e.*, each ORF’s expression level is compared across different ASFV strains) rather than horizontally. The color gradient from blue (lower) to red (higher) signifies the relative expression levels of the ORFs, where a more positive Z-score indicated a relatively higher expression and a more negative Z-score indicated a relatively lower expression. This heatmap is instrumental in visualizing the comparative gene expression patterns of different ASFV strains.

The heatmap of the expression of 131 common ORFs from BA71V, GRG2007, HG2018, and HLJ2018 showed that the overall expression of 131 ORFs in the late stages is relatively higher than that in the early stages (Fig. 3). In addition, the overall expression of HLJ2018 is higher than that of the other three strains. Therefore, from the highest to lowest, the order of overall expression of 131 ORFs is as follows in the early and late stages: HLJ2018 >GRG2007 >HG2018 >BA71V.

### 3.3 Identifying essential common ORFs

**Fig. 4:**
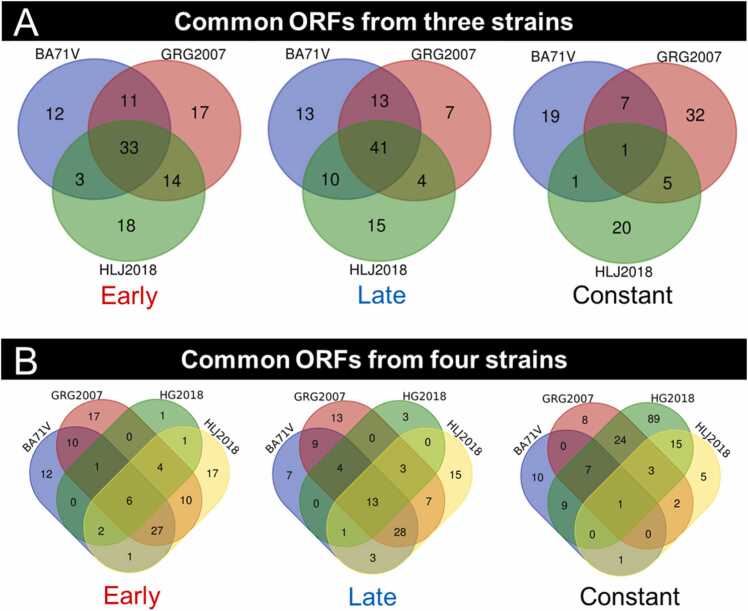
A: Venn diagrams of early, constant, and late genes from BA71V, GRG2007, and HLJ2018. B: Venn diagrams of early, constant, and late genes from BA71V, GRG2007, HG2018, and HLJ2018.

The comparisons of early, late, and constant ORFs among different strains showed that the number of early and late genes in HG2018 was smaller than that in the other strains (Fig. 4). Therefore, the inclusion and exclusion of HG2018 characterized the common ORFs. Exclusion of HG2018, 33, 41, and 1 ORFs were identified as common early, late, and constant genes, respectively (Fig. 4A). Inclusion of HG2018, 6, 13, and 1 ORFs were characterized as early, late, and constant genes, respectively (Fig. 4B). The detailed information is listed in Supplementary Table 2.

**Fig. 5:**
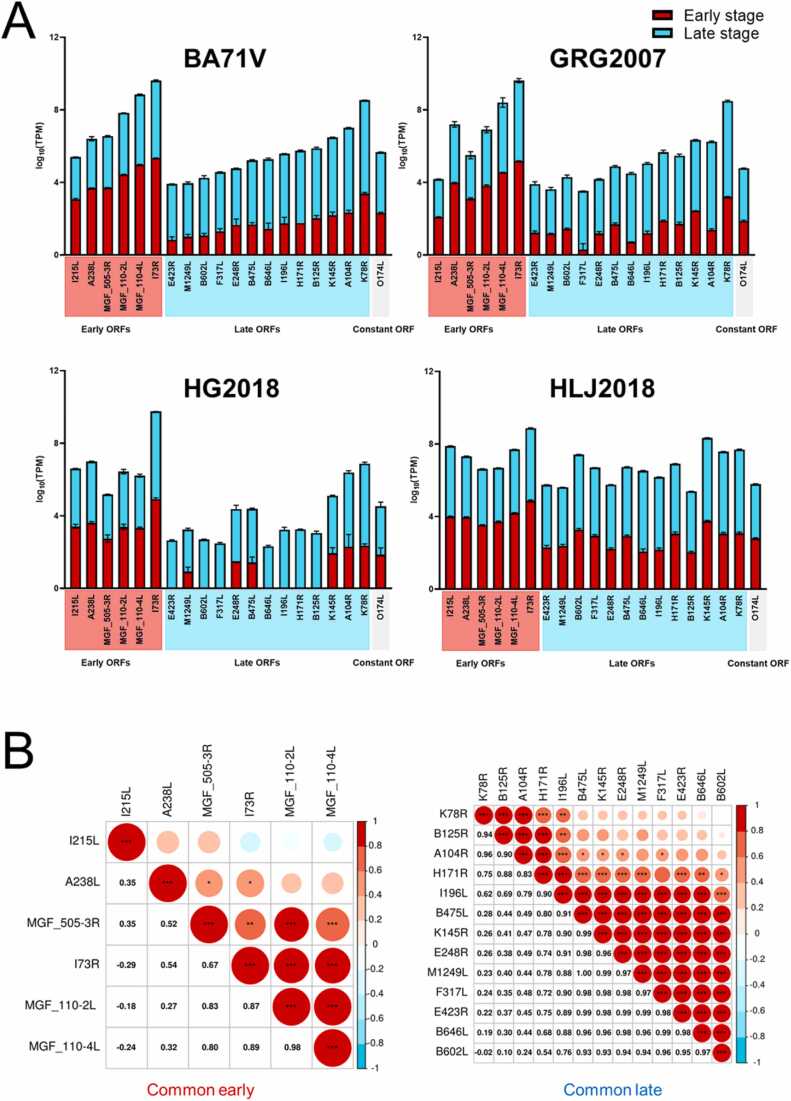
Expression and correlation of essential genes. A: Expression levels (log_10_TPM) of six early genes, 13 late genes, and one constant gene from BA71V, GRG2007, HG2018, and HLJ2018. B: Heatmap of the Pearson correlations of each pair in six early genes and 13 late genes.

The analysis with the inclusion of HG2018 can filter relatively more essential ORFs among all four strains. Therefore, the expression levels of common ORFs were analyzed (Fig. 5).

First, the relative expression levels (log_10_TPM) of each common ORF in the respective strains are presented in Fig. 5A. The results showed that the expressions of I73R and K78R are the highest among the common early and late genes, while I215L and E423R present the lowest expression levels among the common early and late genes, respectively. The overall expression pattern of the shared genes from BA71V is like that from GRG2007 and HG2018. However, in HLJ2018, although I73R exhibits the highest expression in the common early genes, the most highly expressed ORF in the common late genes is K145R rather than K78R.

Furthermore, the correlation heatmap showed that among the common early genes, MGF_110–2L and MGF_110–4L are highly relevant to each other (PCC = 0.98). Among the common late genes, two clusters are observed. The first cluster includes, K78R, B125R, A104R, and H171R (PCC >0.70). The second cluster includes, B475L, K145R, E248R, M1249L, F317L, E423R, B646L, and B602L (PCC >0.70) (Fig. 5B). I196L correlated with both clusters but less strongly than defined.

### 3.4 Identifying unique ORFs

**Fig. 6:**
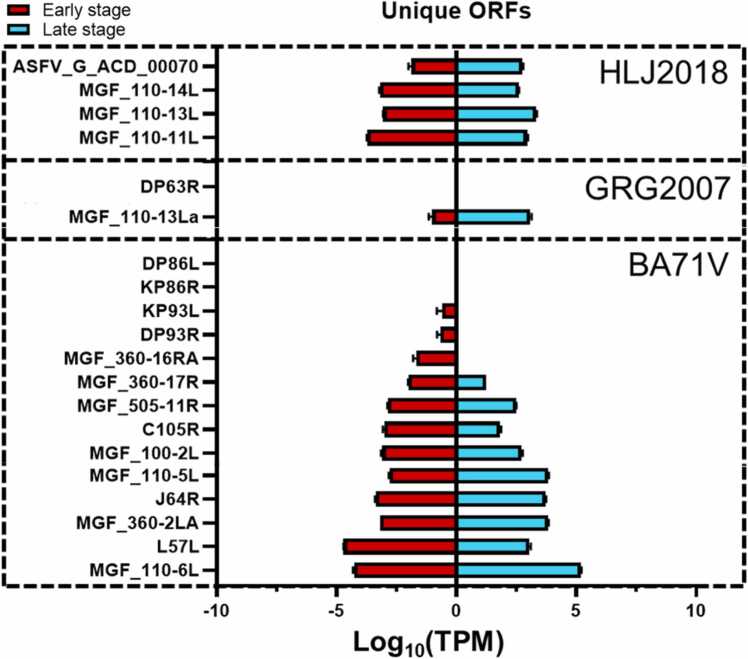
Expression levels (log_10_TPM) of unique genes in HLJ2018, GRG2007, and BA71V.

The unique ORFs in each ASFV strain are shown in Fig. 6. BA71V has 16 unique ORFs, while GRG2007 and HLJ2018 have two and four unique ORFs, respectively. However, HG2018 does not contain any unique ORF.

Among the 14 unique genes in BA71V, DP86L, KP86R, KP93L, and DP93R are not expressed during infection, while MGF_360–16RA is only expressed at the early stage. The rest of the ORFs are expressed at the early and late stages, but they should be considered optional due to the low-virulence of BA71V.

Two unique genes in GRG2007 are DP63R and MGF_110–13La. The former is not expressed during the whole infection, but the latter expresses the early and late stages as a late gene.

Among the four unique genes in HLJ2018, one belongs to the ORF encoding a small protein (ASFV_G_ACD_00070). The other ORFs are MGF_110 members, namely, MGF_110–11L, MGF_110–13L, and MGF_110–14L. All of them are expressed in the early and late stages.

### 3.5 Identifying non-expressed ORFs

The non-expressed ORFs in each strain were identified and summarized in Table 3. In BA71V, four ORFs (KP86L, KP93L, DP93R, and DP86L) were not expressed in either the early or late stages. In addition, these four ORFs are unique in BA71V.

**Table 3:** Non-expressed ORFs*.

Strain	Non-expressed ORFs
BA71V	**KP86R**, **KP93L**, **DP93R**, **DP86L**
GRG2007	DP60R, ASFV_G_ACD_01990, ASFV_G_ACD_00160, ASFV_G_ACD_00360, D129L, DP79L, D339L, D1133L, D117L, ASFV_G_ACD_01760, **DP63R**
HLJ2018	ASFV_G_ACD_00360, ASFV_G_ACD_01020, ASFV_G_ACD_01760
HG2018	DP60R, ASFV_G_ACD_01990, ASFV_G_ACD_00090, ASFV_G_ACD_00160, ASFV_G_ACD_00190, ASFV_G_ACD_00210, ASFV_G_ACD_00360, X69R, ASFV_G_ACD_00520, ASFV_G_ACD_01020, S273R, H233R, QP383R, ASFV_G_ACD_01870, DP71L

*: ORFs in bold also refer to unique genes in each strain.

In GRG2007, eleven ORFs were identified as non-expressed ORFs, four of which are ORFs encoding small proteins. Among the other non-expressed ORFs, DP63R is a unique ORF in GRG2007.

In HG2018, 15 ORFs were identified as non-expressed ORFs, nine of which are ORFs encoding small proteins. The other non-expressed ORFs were DP60R, X69R, S273R, H233R, QP383R, and DP71L.

**Fig. 7:**
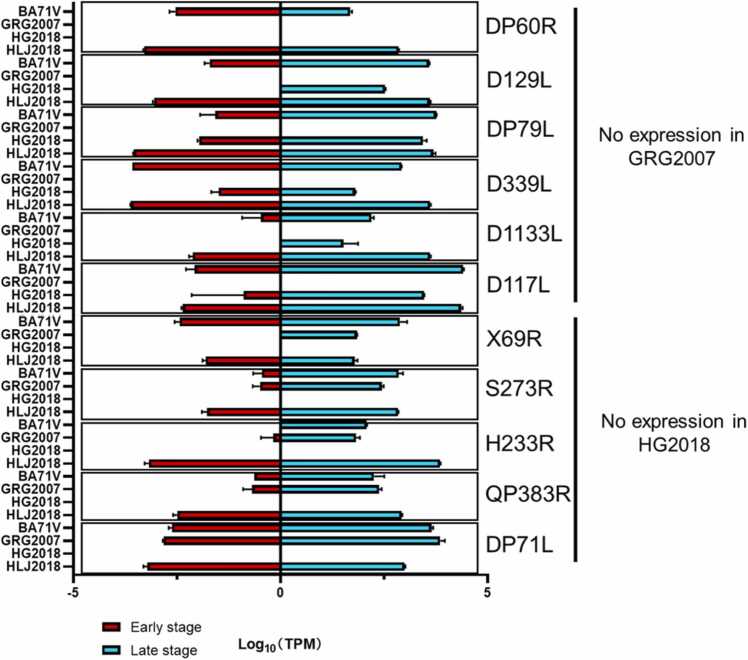
Expression levels (log_10_TPM) of non-expressed open reading frames in different strains.

In HLJ2018, three ORFs were identified as non-expressed ORFs. All of them are ORFs encoding small proteins. The expression levels of these non-expressed ORFs are summarized in Fig. 7.

### 3.6 Effects caused by ORF deletions

The comparison of ORFs among different strains and their deletion effects are summarized in Supplementary Table 3. Single deletions of MGF_110–9L, MGF_360–9L, MGF_505–7R, A104R, A137R, A151R, B119L, DP96R, E184L, I177L, and I226R resulted in strong attenuation of highly virulent strains. Ten mutants have been reported with strong attenuation effects in the case of multi-deletion. The most frequently selected ORFs involved in more than five mutants as multi-deletion targets were MGF_360 (−12L, −13L, −14L), MGF_505 (−1R, −2R, −3R), and EP402R.

### 3.7 Structure and interaction prediction of essential genes

**Fig. 8:**
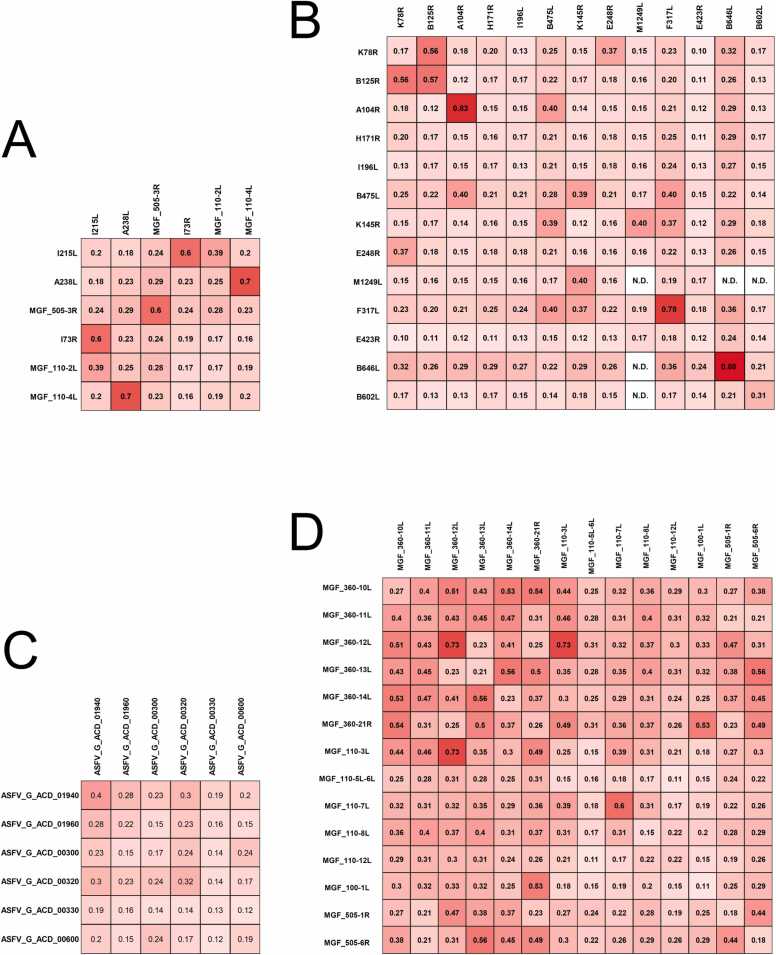
Interaction scores of six early genes (A), 13 late genes (B), six short genes encoding small proteins (C), and 14 MGF (D). The number in every cell represented the ranking_confidence score for each prediction. N. D. means AlphaFold2 returned no complex structure.

**Fig. 9:**
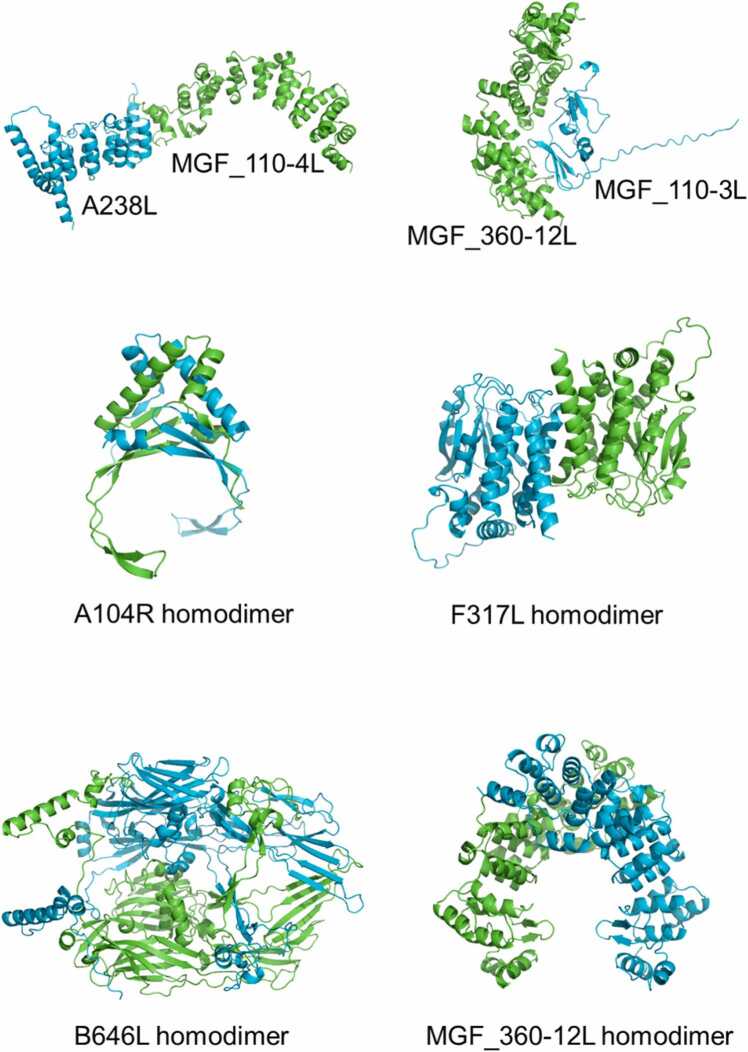
Structures of protein complexes predicted by AlphaFold2 with a confidence_score ≥ 0.7.

The structures of six early genes, 13 late genes, six short genes encoding small proteins, and 14 MGFs were predicted using AlphaFold2, along with their potential interactions. The structural files can be found in **Supplementary File 3**, and their ranking_confidence scores are presented in Fig. 8. Scores ≥ 0.7 indicated a higher likelihood of interaction and their structures were shown in Fig. 9. Among the six early genes, the interaction between A238L and MGF_110–4L showed a high score (0.7) (Fig. 8A). In the 13 late genes, no hetero-protein interactions met the threshold of a score ≥ 0.7; however, A104R, F317L, and B646L were observed to have self-interaction scores exceeding 0.7, suggesting that they may function as dimers in a biological environment (Fig. 8B). No interaction among the six small proteins was found with scores ≥ 0.7 (Fig. 8C). Among the 14 MGFs, heterodimers with scores ≥ 0.7 were identified between MGF_360–12L and MGF_110–3L, and a homodimer was identified for MGF_360–12L (Fig. 8D).

## 4 Discussion

### 4.1 Potential essential ORFs from 131 common ORFs

A total of 131 common ORFs were characterized in all strains’ common early/late/constant genes. The results showed that all the viral strains identified at least six early genes, 13 late genes, and one constant gene to maintain their propagation (Fig. 4).

Among the six early genes, MGF_110–2L and MGF_110–4L were highly related (Fig. 5B). MGF proteins are widely distributed in ASFVs, but their functions have yet to be well characterized and verified. However, predictions have shown that MGF_110 proteins contained cysteine-rich motifs ideal for oxidizing environments. Furthermore, MGF_110–2L and MGF_110–4L have a signal peptide. They are secreted in the extracellular space and endoplasmic reticulum (ER) [28], suggesting that MGF_110–2L and MGF_110–4L are related to early infections. No scientific work has targeted MGF_110–2L, but one article has compared the genomic differences between the highly virulent ASFV Lisboa60 (L60) and the low-virulent ASFV NH/P68 (NHV). The results suggested that the insertion of 4458 bp after MGF_110–2L in NHV is a critical difference from L60, which indicated that an incorrect expression of MGF_110–2L may cause an attenuation in the highly virulent strain [29]. For MGF_110–4L, it was assumed that it is not essential for virulence in pigs [30]. For example, a strain from Congo (Uvira B53) does not possess MGF_110–4L but is still virulent [31]. However, the high expression of MGF_110–4L in BA71V, GRG2007, HG2018, and HLJ2018 at the early stages warrants further investigations.

Two clusters were observed in the correlation analysis in the 13 late genes (Fig. 5B). Cluster I contained K78R, B125R, A104R, and H171R. In Cluster I, K78R and A104R are reported as structural proteins with DNA-binding activity (Table 4) [32]. K78R encodes a DNA-binding protein named p10. This ORF has been selected as a target for developing a loop-mediated isothermal amplification method for the early diagnosis of ASF [33]. A longitudinal serological study with pigs infected with the attenuated NH/P68 strain showed a poor antibody response to K78R [34], and no published report demonstrating the effects of K78R deletion on ASFV (Supplementary Table 3). However, A104R is a histone-like structural protein involved in viral transcription, DNA replication, and genome packaging, indicating a potential for vaccine and drug development [35]. The A104R protein is one of twelve viral proteins implicated in serological immunity in pigs. The presence of antibodies against this protein showed an effective immune response that might be involved in protection [32]. Cluster I included ORFs for hypothetical proteins (B125R, H171R) with unidentified functions (Table 4). In recent publications, H171R was predicted to be a structural protein stabilizing virus particles [36], but its specific role and mechanism remain unclear. Therefore, B125R, H171R, and their highly relevant histone-like protein, A104R, could be potential targets for further investigations.

**Table 4:** Common ORFs and functions.

Stage	ORF	Function	Category
Early	MGF_110–2L	HCF-1 binding motif	Multi-Gene family
	MGF_110–4L	BRCT phosphopeptide ligands	Multi-Gene family
	MGF_505–3R	WDR5 WD40 repeat (central)-binding ligand	Multi-Gene family
	A238L	IkB-like protein	Immunosuppression
	I215L	Ubiquitin-conjugation enzyme	mRNA modification
	I73R	Hypothetical protein	Unknow
Late	B602L	Chaperone	Assembling and releasing
	B646L	Structural protein p72	Capsid
	E248R	Putative transmembrane domain containing protein	Inner Envelope
	K78R	Structural protein p10; DNA-binding activity	DNA binding proteins
	A104R	Histone-like structural protein IHF-like DNA-binding protein	DNA binding proteins
	I196L	Putative signal peptide	Unknow
	M1249L	Ubiquitin-like domain containing protein	Unknow
	B125R	E2 early regulatory protein	Unknow
	E423R	Hypothetical protein	Unknow
	F317L	Hypothetical protein	Unknow
	K145R	Hypothetical protein	Unknow
	H171R	Hypothetical protein	Unknow
	B475L	Hypothetical protein	Unknow
Constant	O174L	DNA polymerase beta-like protein	Base excision repair

Cluster II contained more ORFs, including B475L, K145R, E248R, M1249L, F317L, E423R, B646L, and B602L. In this cluster, the most frequently reported ORFs were B646L and B602L. B646L encoded the structural protein p72, which folds as a trimer and assembles into an icosahedral capsid. This capsid is the major component of the outermost protein shell of the virus particle [37]. B602L encodes a non-structural protein that works as a molecular chaperone. It has been reported that the repression of protein B602L synthesis inhibited the proteolytic processing of p72 and the formation of capsids [38].

Consequently, B602L has attracted attention as a target to produce monoclonal antibodies. In addition to B646L and B602L, E248R is predicted to be a structural protein located in the inner envelope of the virus particle [39]. Such inner viral membrane proteins might be implicated in the cell—virus fusion step during infection, and the target receptors in the host cell are cellular endosomal proteins, such as Niemann-Pick C type 1 (NPC1) and lysosomal membrane proteins (Lamp−1 and −2) [40]. Recently, the E248R protein inhibited the cGAS-STING pathway by suppressing the expression of STING protein in HEK293 cells [41]. Furthermore, the E248R gene was selected as the target for establishing a rapid, specific, and sensitive diagnosis of ASF infections [42]. However, in Cluster II, some more ORFs have not been functionally revealed (Table 4). M1249L was predicted to be a ubiquitin-like domain-containing protein, but the others (E423R, F317L, K145R, and B475L) were all hypothetical proteins with uncharacterized functions. Until recently, some of the unknown proteins have drawn attention to research. For example, overexpressing F317L has been reported to promote ASFV replication, and the knockdown of F317L expression suppressed ASFV replication [43]; the deletion of K145R from GRG2007 had a mild attenuating effect [44]. Nevertheless, according to our study, they are all highly expressed in the late stages, and their expression levels were highly relevant to B646L/B602L and E248R. Therefore, they can be potential targets for further analysis.

In addition to the two clusters, one ORF, I196L, was identified to be weakly correlated to each cluster. Another group identified I196L as an essential gene expressed in the late stage after infection by HLJ2018 [45].

Our DESeq2 study identified one common constant gene, O174L that was reported to encode a reparative DNA polymerase that belongs to family X of DNA polymerases, such as cellular DNA polymerase β. Therefore, it has been designated as ASFV Pol X [46]. As a result of the deletion of O174L from the BA71V genome, the mutant virus does not reproduce in swine macrophages when multiple rounds of DNA replication occur [47]. Recently, O174L has been used as the genetic marker to track the virus originating from Polish ASFV strains [48].

Nevertheless, judging from the TPM data, A137R was constantly expressed in four strains (Supplementary Table 3) and has been reported as a promising target for vaccine development since the A137R gene deletion results in attenuating HLJ2018 [49].

### 4.2 Potentially essential ORFs from unique ORFs in virulent strains

Most of the ORFs present in virulent strains were MGF members, including MGF_100 (1L), MGF_110 (3L, 5–6L, 7L, 8L, and 12L), MGF_360 (10L, 11L, 12L, 13L, and 21R), and MGF_505 (1R and 6R) (Table 2). Those MGF members were absent in the avirulent strain BA71V and OURT88/3 (Fig. 1B). Structure prediction of those MGF members showed that they have intensive ankyrin repeat-containing domains, which indicated that they could interact with lipids or lipid bilayers in the extracellular or cytoplasmic area. Information regarding the functions of MGF members is still limited. However, since many attenuated ASFV strains are based on deleting MGF members, intensive investigations revealed their functionality, especially those MGFs only present in virulent strains.

It is worth noting that the alignment of amino acid sequences of MGF_110–5–6L, MGF_110–5L, and MGF_110–6L revealed that an ER retained signal sequence (KDEL) at the C-terminus of MGF_110–6L from BA71V and Kenya05. At the same time, MGF_110–5L and MGF_110–5–6L (present in highly virulent strains) do not have this ER retention signal. Besides, the KDEL sequence can change to an unusual ER retention sequence KEDL (MGF_110–6L from OURT88/3). This sequence variant localized MGF_110 proteins at post-ER-pre-Golgi structures by interacting with the KDEL receptor that controls the distribution of lumenal ER proteins within pre-Golgi membrane compartments [50]. MGF_110–6L tends to be less toxic when retained in the ER or post-ER-pre-Golgi structures than fused with MGF_110–5L (Supplementary Figure 1).

### 4.3 Potentially essential ORFs from non-expressed ORFs

Each strain’s genomic sequence contained ORFs that were not expressed in mRNA. These ORFs were generally considered non-expressed ORFs with little influence on virulence.

DP60R [51], D129L, DP79L, D339L, D1133L [52], and D117L were non-expressed ORFs in GRG2007. However, all of them had a high expression level in HLJ2018. Among them, D339L is a component of the virus RNA polymerase. Although the rest of the ORFs have been sequenced, the functions of their putative protein products remain unknown.

Furthermore, X69R, S273R, H233R, QP383R, and DP71L were non-expressed ORFs in HG2018, but they all showed high expression levels in BA71V, GRG2007, and HLJ2018. X69R is a nonessential gene because its deletion from GRG2007 does not affect virulence in swine [53]. S273R is an essential protease that digests polyproteins (the precursor of core shell) of ASFV [3], [54], which has become a target for vaccine and medicine discovery for GRG2007.

However, in certain virus strains, the expression levels of S273R are very low or even undetectable. For example, Olesen et al. (2021) reported that in pigs infected with the POL/2015/Podlaskie virus strain, S273R is expressed at deficient levels after 6 h of infection [55]; Lv et al. (2022) reported that the expression levels of S273R are low or sometimes undetectable during 6–48 h of infection with the HuB20 virus strain to PAMs [56]. The amount of mRNA present does not solely determine the amount of protein produced, as it can be influenced by protein stability and mRNA translational efficiency.

Deleting QP383R and QP509L from the CN/GS/2018 strain resulted in complete viral attenuation in swine [57]. DP71L restores protein synthesis by recruiting protein phosphatase 1 to dephosphorylate eukaryotic initiation factor 2α (eIF2α) and producing viral proteins by hijacking the cellular protein translational machinery [45], [58]. H233R is the only ORF whose function remains unclear.

### 4.4 Potentially essential ORFs encoding small proteins

The ORFs beginning with the label “ASFV_G_ACD” represent small proteins (Table 5). The sequence of tone small protein in the NCBI entries starts from valine. However, not methionine (Table 5) because it uses an alternative initiation codon, “GTG”, and the function of this small protein, is currently limited. These small proteins are only annotated in highly virulent strains (HG2018, GRG2018, and HLJ2018) but not low-virulence strains (OURT88/3 and BA71V). Therefore, it was essential to analyze the expression of those small proteins. Our bioinformatic analysis showed that although the presence and expression levels of “ASFV_G_ACD” ORFs varied among the three strains, the following “ASFV_G_ACD” ORFs were expressed in all three strains: ASFV_G_ACD_00300, 00320, 00330, 00600, 01940, and 01960 (Supplementary Table 3). Currently, insufficient experimental data support their significance in the virulence of ASFV. However, they have shown significant potential in distinguishing between virulent and avirulent strains.

**Table 5:** ORFs encoding small proteins.

ORF	Amino acid sequence of the encoded small proteins	Length
ASFV_G_ACD_00070	VANALLASACKVDGCMFILLKKNQIIG	27
ASFV_G_ACD_00090	MATCEQVAARQQIAVYQQIAVYQQQLIISKCCLWVSQ	37
ASFV_G_ACD_00120	MFYREKVFLAYECTWFRTLKKNKRNYLILAVIFFQLATSQVTKELHCSFILRKRLNFGQKKSYLIKNPHRSFFS	74
ASFV_G_ACD_00160	MHQMNLLDQDFIDVSMTIPGLRELIRVTGTLVFHAVLHIKPA	42
ASFV_G_ACD_00190	MFFFSYFATGNIKWWPVHLFNKKILTNLLLSLSLTYPVTIL	41
ASFV_G_ACD_00210	MAATYKLQLMGCNTATYRLHLIDRDSKGMKTLPSIQNLSFNPDNVSVYEKKFFLLMYEFLYES	63
ASFV_G_ACD_00240	MLCPNGTCHFKKDLNFATAKKNPVCIFLIHIIIEVL	36
ASFV_G_ACD_00270	MVYNFNTLYDVGADPRNNIRNFKVVFLCYIQNLNFFL	37
ASFV_G_ACD_00300	MFIIHTNIMNFFCNIKLFLYHFRSIHKKNILKLFNFIE	38
ASFV_G_ACD_00320	MFNISSILIQGSVMILILLLWIINENFTDAGGNNFNHTVLM	41
ASFV_G_ACD_00330	MLKCLKIIVHQCCKFDQNLISHKKGTHQHCSFKFHDV	37
ASFV_G_ACD_00350	MFDSLLPTITGGGGGSLIALALLWFADYYVEFIEARLDSNITAV	44
ASFV_G_ACD_00360	MIKNRCIAQYSFGTSLEMIQATMITMYNSIVIFFFCNV	38
ASFV_G_ACD_00520	MFPTDVLKRKHLFTKKNINFTGILMYDKTVVHWFEISKTI	40
ASFV_G_ACD_00600	MSGFMNSLRKCIGNINSHLEGFMRTYLLRIIRKIKPTTQPSIEDDHYKCL	50
ASFV_G_ACD_01020	MKKKDAYEQAKAYNILNVAFLLPVMALPKDMLLRIFPKELVQTLLSQEGQRD	52
ASFV_G_ACD_01760	MWKVNDQGFLNISVTGTKFNLIAITGKLGFYTDPPSHLIIMPLKFFPVHKFSKNEPNKKQKRFIYF	66
ASFV_G_ACD_01870	MYFQQTRSILIKNDAVFILNLGLKFKEVFINYNHGNFFSNCLCKK	45
ASFV_G_ACD_01940	MDIWYLKYVIAYILLLTMLVIGLIYRIIVLIYRSIQAQKNVVCNNALALFAM	52
ASFV_G_ACD_01960	MVAFKNIKKTLSFRQQQIVCRRPQTIFRVFCTIKYFFWSGLLL	43
ASFV_G_ACD_01980	MIKKKNYFFGQHLFVRIVDKSAGNNNIYNFFLGEKNNYVTSYIPIGDLSPSESSVIKNNFVSMR	64
ASFV_G_ACD_01990	MNIYLVWFLYILLGNLILAVIYCVIDEVVCDNIHIKKNVAAPEMPRRF	48

### 4.5 Potential interaction prediction of essential proteins

Complete structural predictions for all ORFs from GRG2007 have been reported [59]. Therefore, in this study, we focused on predicting interactions among the essential gene products, including six early genes, 13 late genes, six short genes encoding small proteins, and 14 MGFs. Among the six early genes, a high interaction score was observed between A238L and MGF_110–4L (Fig. 8A). Based on transcriptomic data, their expression levels were not highly correlated (Fig. 5B), but were identified as early expressed genes, suggesting a high likelihood of interaction. A238L exhibited weak attenuation when knocked out from Kenya-IX-1033 [60]. The functions of MGF_110–4L remain unknown, but its sequence contains an ER retrieval sequence (Supplementary Table 3). Although the 13 late genes were classified into two clusters with high expression correlation based on transcriptomic data (Fig. 5B) the potential for protein interactions among them was low (Fig. 8B). No protein interactions were identified among the six short genes encoding small proteins (Fig. 8C). However, among the 14 MGFs, a high interaction potential was observed between MGF_360–12L and MGF_110–3L (Fig. 5C and D). The function of MGF_110–3L remains unclear, but MGF_360–12L was identified as inhibiting type I interferon production and has been targeted for gene knockout in several studies (Supplementary Table 3).

### 4.6 Comparison of transcriptomic and proteomic data

This study conducted genomic and transcriptomic analyses focusing on multiple different ASFV strains, identifying crucial genes/proteins that play significant roles in viral invasion and interaction with the host. Our results highlighted six early genes (MGF_110–2L, MGF_110–4L, I73R, I215L, MGF_505–3R, and A238L), 13 late genes (B475L, B602L, B646L, B125R, H171R, E423R, E248R, I196L, A104R, F317L, K78R, K145R, and M1249L), six short genes encoding small proteins (ASFV_G_ACD_01940, ASFV_G_ACD_01960, ASFV_G_ACD_00300, ASFV_G_ACD_00320, ASFV_G_ACD_00330, and ASFV_G_ACD_00600), and 14 MGFs (MGF_360–10L, MGF_360–11L, MGF_360–12L, MGF_360–13L, MGF_360–14L, MGF_360–21R, MGF_110–3L, MGF_110–5–6L, MGF_110–7L, MGF_110–8L, MGF_110–12L, MGF_100–1L, MGF_505–1R, and MGF_505–6R) as being highly significant.

In contrast, Ros-Lucas et al. reported the computational proteome analysis to identify potential antigenic epitopes within the ASFV Georgia 2007/1 strain [61]. They analyzed ASFV (Georgia 2007/1) protein sequences to identify specific epitopes of interest for a prospective vaccine ensemble. They reported that 13 ORFs contained B cell epitopes (E402R, KP177R, E183L, B646L, D117L, MGF_110–9L, B438L, B318L, B169L, C257L, B475L, E146L, and M1249L), 13 ORFs contained CD4^+^ T cell epitopes (B117L, ASFV_G_ACD_01990, EP424R, ASFV_G_ACD_01940, B646L, C147L, D1133L, E402R, ASFV_G_ACD_01870, B66L, MGF_360–21R, C62L, and A118R), and six ORFs contained CD8^+^ T cell epitopes (D205R, D339L, EP1242L, F334L, P1192R, and G1340L).

Common elements, such as ASFV_G_ACD_01940, B646L, B475L, and M1249L, are significant in both studies, suggesting their potential significance in ASFV biology and immunogenicity.

In our transcriptomic analyses, ASFV_G_ACD_01940 is identified as one of the six short genes, indicating its potential role in the viral structure or function. However, in proteomic analysis, ASFV_G_ACD_01940 is recognized as containing CD4^+^ T cell epitopes. Besides, B646L is classified as one of the 13 late genes, indicating its potential role in the later stages of the virus replication cycle. In contrast, Ros-Lucas et al. identified B646L as containing B cell and CD4^+^ T cell epitopes, suggesting its potential as a target for immune response and vaccine development. Similarly, B475L and M1249L are part of the 13 late genes and are identified as containing B cell epitopes. This dual recognition underscores the potential multifunctionality of B475L in ASFV’s life cycle and its possible role as an immunogenic target. These shared findings between the two studies provided valuable insights into the complexity of ASFV, highlighting their potential significance in eliciting an immune response.

## 5 Conclusions

Among the 131 common ORFs from four ASFV strains, six early ORFs were identified as early genes, whereas 13 were late genes. In the later stage of infection, two clusters with high correlation were observed. In addition, six “ASFV_G_ACD” members were expressed in all highly virulent strains, and 14 MGF members were absent in the low-virulent strains. The information obtained will be a potential guideline for revealing the virulence mechanism in different strains and exploring new therapeutic targets for combating ASF.

## Author statement

During the preparation of this work, the authors only used basic tools (DeepL and Zotero) for checking grammar, spelling, and references. No AI tools or services were used. The authors take full responsibility for the content of the publication.

## CRediT authorship contribution statement

**Peng Lu**: Conceptualization, Methodology, Data curation, Formal analysis, Visualization, Roles/Writing – original draft; Writing – review & editing. **Jiaqiao Zhou**: Data curation, Formal analysis, Writing – review & editing. **Sibo Wei**: Data curation, Formal analysis. **Konosuke Takada**: Data curation, Formal analysis. **Hayato Masutani**: Data curation, Formal analysis. **Suguru Okuda**: Investigation. **Ken Okamoto**: Investigation. **Michio Suzuki**: Investigation. **Tomoya Kitamura**: Writing – review & editing. **Kentaro Masujin**: Writing – review & editing. **Takehiro Kokuho**: Funding acquisition, Writing – review & editing. **Hideaki Itoh**: Funding acquisition, Writing – review & editing. **Koji Nagata**: Conceptualization, Supervision, Funding acquisition, Writing – review & editing.

## Declaration of Competing Interest

The authors declare that they have no known competing financial interests or personal relationships that could have appeared to influence the work reported in this paper.
